# Case report: A rare complication after the implantation of a cardiac implantable electronic device: Contralateral pneumothorax with pneumopericardium and pneumomediastinum

**DOI:** 10.3389/fcvm.2022.938735

**Published:** 2022-08-18

**Authors:** Shao-Wei Lo, Ju-Yi Chen

**Affiliations:** Department of Internal Medicine, College of Medicine, National Cheng Kung University Hospital, National Cheng Kung University, Tainan, Taiwan

**Keywords:** contralateral pneumothorax, pneumopericardium, pneumomediastinum, cardiac implantable electronic device, pacemaker

## Abstract

Cardiac implantable electronic devices (CIED) including pacemakers (PM), implantable cardioverter defibrillators (ICD), and cardiac resynchronized therapy (CRT) have become the mainstay of therapy for many cardiac conditions, consequently drawing attention to the risks and benefits of these procedures. Although CIED implantation is usually a safe procedure, pneumothorax remains an important complication and may contribute to increased morbidity, mortality, length of stay, and hospital costs. On the other hand, pneumopericardium and pneumomediastinum are rare but potentially fatal complications. Accordingly, a high degree of awareness about these complications is important. Pneumothorax almost always occurs on the ipsilateral side of implantation. The development of contralateral pneumothorax is uncommon and may be undetected on an initial chest radiograph. Contralateral pneumothorax with concurrent pneumopericardium and pneumomediastinum is much rarer. We describe a rare case of concurrent right-sided pneumothorax with pneumopericardium and pneumomediastinum after left-sided pacemaker implantation and highlight the risk factors, management, and possible ways to prevent the complications.

## Introduction

Cardiac implantable electronic devices (CIED) including pacemakers (PM), implantable cardioverter defibrillators (ICD), and cardiac resynchronized therapy (CRT) have become the mainstay of therapy for many cardiac conditions, consequently drawing attention to the risks and benefits of these procedures (1, 2). Although CIED implantation is usually safe, pneumothorax has been reported to occur in 0.51–2.24%, however this reported incidence may have been overestimated (3). In recent years, many CIED implantations have moved to outpatient settings. Outpatient CIED implantations are not included in the National Inpatient Sample (NIS) database, which is the largest publicly available all-payer inpatient care database in the United States. Since the subgroup of outpatients who developed CIED-associated pneumothorax and were later hospitalized (included in the NIS, while outpatients without pneumothorax were not), the NIS database incidence of pneumothorax may have been artificially elevated (an accurate numerator with a falsely low denominator) (3). Furthermore, it seems likely that improved medical knowledge and the development of safer procedures, would have reduced the incidence of pneumothorax over time (3). Nevertheless, pneumothorax remains an important complication of CIED implants and may contribute to increased morbidity, mortality, length of stay, and hospital costs, especially when a chest tube is required (4–7). On the other hand, pneumopericardium and pneumomediastinum are rare but potentially fatal complications (8). Accordingly, a high degree of awareness about these complications is important.

Pneumothorax almost always occurs on the ipsilateral side of implantation. The development of contralateral pneumothorax is uncommon and may be undetected on an initial chest radiograph (9, 10). Contralateral pneumothorax with concurrent pneumopericardium and pneumomediastinum is much rarer (11). We describe a rare case of concurrent right-sided pneumothorax with pneumopericardium and pneumomediastinum after left-sided pacemaker implantation and highlight the risk factors, management, and possible ways to prevent these complications.

## Case presentation

A 76-year-old man underwent dual-chamber permanent pacemaker (PPM) implantation due to sick sinus syndrome. He was 173 cm in height, 59 kg in weight, and had a body mass index (BMI) of 20 kg/m^2^. He had a past medical history of heavy smoking, paroxysmal atrial fibrillation, coronary atherosclerosis, and bilateral pulmonary emphysema.

A dual-chamber pacemaker (BIOTRONIK EVIA DR) was inserted using active fixation leads (Biotronik Solia S60, Biotronik Solia S53) through the left subclavian vein into the right ventricular outflow tract (RVOT) and the right atrial appendage respectively. At implantation, the parameters of the pacemaker were satisfactory. The atrial lead pacing threshold was 0.6 V at 0.4 ms and the impedance was 532 Ω. The sensed P wave was 2.3 mV. The pacing threshold of the ventricular lead was also appropriate measuring 0.4 V at 0.4 ms and the impedance 661 Ω. The sensed R wave was 11.2 mV.

**Figure 1:**
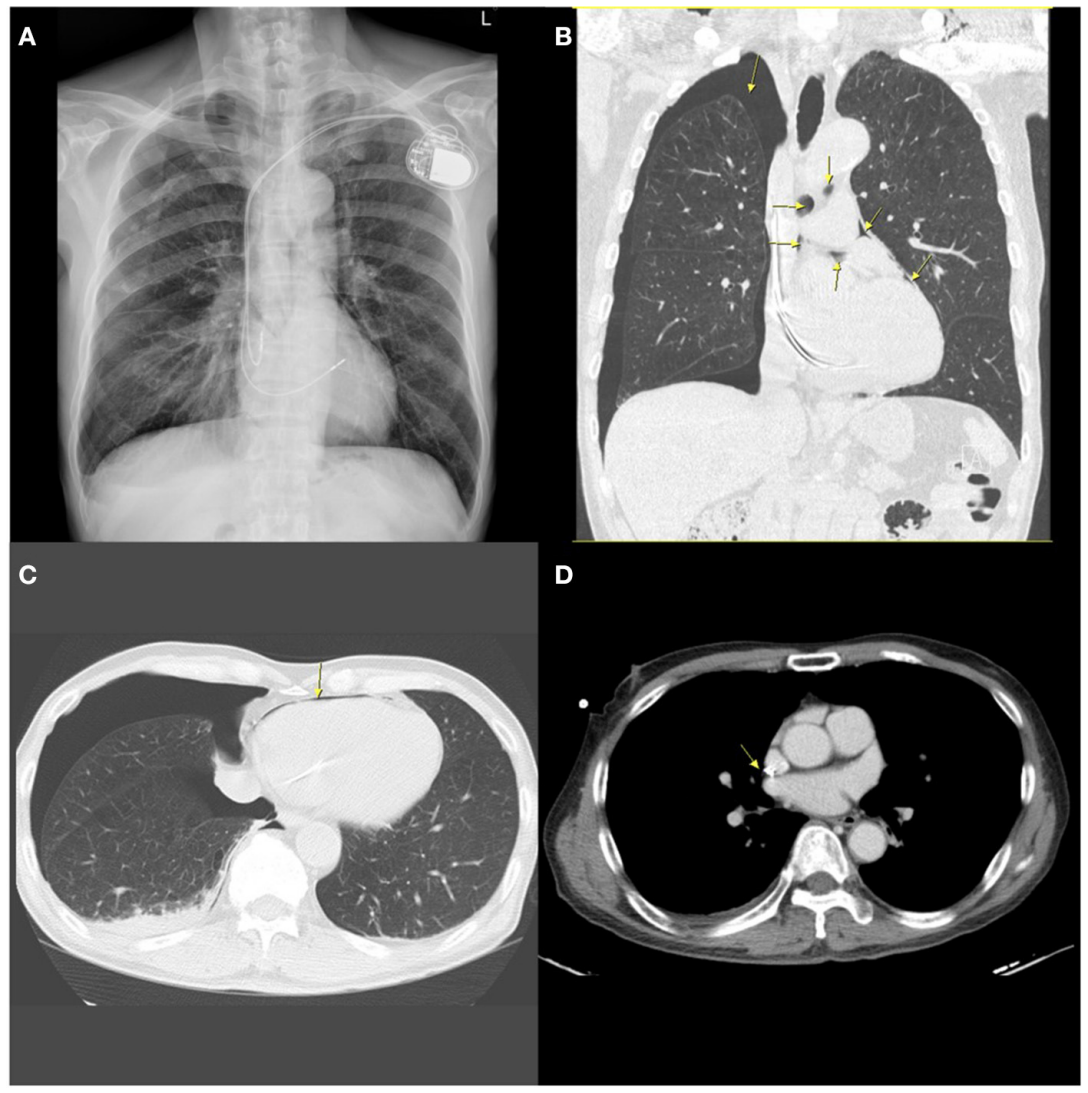
A chest radiograph performed 5 h after implantation showing contralateral pneumothorax, pneumopericardium, and pneumomediastinum. **(A)** Posterior-anterior (PA) chest X-ray demonstrating contralateral pneumothorax and mild pneumomediastinum. **(B)** Coronal chest non-contrast computed tomography (CT) scan image showing right-sided pneumothorax, pneumomediastinum, and pneumopericardium (arrows). **(C)** Horizontal CT image showing pneumopericardium (arrow) and pneumothorax. **(D)** Horizontal slices of CT scan suggesting possible extrusion of the atrial lead through the right atrium (arrow).

The implantation procedure was completed uneventfully. Two h after the implantation, the chest radiographs revealed acceptable lead positions and no evidence of pneumothorax. There was no pericardial effusion on echocardiography. About 5 h after the procedure, the patient suddenly reported dyspnea, severe headache, neck stiffness, and shoulder pain. A chest X-ray (CXR) revealed a 3.5 cm right-sided apical pneumothorax as well as small amounts of gas as linear or curvilinear lucencies in the mediastinum, indicating pneumomediastinum. Non-contrast computed tomography (CT) of the chest showed bilateral emphysema, right-sided pneumothorax with pneumopericardium and pneumomediastinum, a small right-sided pleural effusion, and the atrial lead crossing the cardiac contour, suggesting lead perforation through the pericardium and directly into the pleural cavity (Figure 1).

The patient's symptoms improved significantly after receiving high-flow oxygen through a nasal cannula. Nevertheless, the follow-up CXR showed no improvement. The patient, therefore, underwent insertion of a small-bore pigtail chest drain on the 3rd day, evacuating more than 80 ml of air. Serial CXRs showed significant improvement in the pneumopericardium and gradual resolution of the pneumothorax over the next few days. The electrocardiogram (ECG) showed no abnormal changes suggesting lead displacement, and interrogation of the pacemaker parameters remained fine with no significant alterations in the pacing thresholds, sensing or impedance. It was decided to leave the atrial lead in place.

During several days of in-hospital observation, the patient remained stable with no breathing difficulties, pleuritic chest discomfort, or pericardial signs and symptoms. The pigtail was then removed on day 6. The CXR verified the complete resolution of the pneumothorax and pneumopericardium before the patient was discharged on day 8. Since discharge, we have followed up on his signs and symptoms, pacemaker parameters, and ECG. The patient has remained stable for 15 months, and the pacemaker parameters and ECG have demonstrated no abnormalities.

## Discussion

### Mechanisms of ipsilateral/contralateral pneumothorax with concurrent pneumopericardium/pneumomediastinum

**Figure 2:**
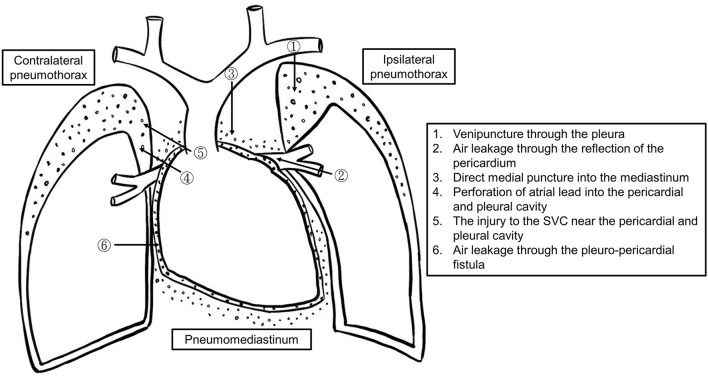
The schematic diagram of the mechanisms of pneumothorax, pneumopericardium, and pneumomediastinum formation.

Ipsilateral pneumothorax is commonly caused by needle penetration of the pleura during venous access (Figure 2) (5, 10, 12–14). Concurrent pneumopericardium and pneumomediastinum can occur when the leaking air passes through the lung parenchyma along the perivascular sheaths to the hilum and the mediastinum (15, 16). At its reflection enclosing the ostia of the pulmonary veins, the pericardium is most fragile, and air can thus pour into the pericardial cavity.

There are two potential causes of contralateral pneumothorax and pneumopericardium. One is that the active fixation lead protrudes through the right atrium injuring the pericardium and right pleura (16). Another one could be incidental venous puncture of the right pleura while using the Seldinger technique to insert a dilator and sheath (17). An injury to the superior vena cava (SVC) near the pericardium could allow air (blood and/or intravenous fluid) to leak into the pericardial space through a congenital pericardial defect or tiny pleuro-pericardial fistulas (11).

### Risk factors for atrial lead protrusion and venous perforation

Complications from pacemaker implantation are 30% more likely in females than in males (3). The incidence of both venipuncture related injury and cardiac perforation is higher in patients with bullous emphysema, chronic obstructive pulmonary disease (COPD), congenital defects such as persistent left superior vena cava, age >80 years, Caucasian ethnicity, steroid treatment within 7 days, anticoagulant and antiplatelet therapy, urgent surgery, low BMI (<18.5), or agitation (1, 8, 15, 18–20). Prior procedures, operation (such as sternotomies), trauma, or irradiation therapy in the affected area, clavicle/chest deformity, and previous fractures, are all significant risk factors. Difficult or lengthy procedures, large diameter (≥12 French) sheaths, more than one attempt at venipuncture, implantation of multiple electrodes, and a dual-chamber device (versus a single chamber device), are all linked to a higher risk for complications (1, 12, 14, 18, 21).

In comparison to passive fixation leads, active fixation leads provide several benefits, such as simple fixation, the capacity to be deployed at alternate pacing sites with ideal pacing and sensing parameters, decreased rates of dislodgement, and they are easier to extract (22). Because of these advantages, they are more frequently employed. Nevertheless, active fixation and over-screwing increase the risk of perforation (8, 16, 17, 20). Lead and helix design also play a role, particularly in the case of magnetic resonance imaging (MRI) compatible leads due to their greater diameter and stiffness (20). As a result, these active fixation leads should be implanted cautiously. Anatomic variations, such as multilobed or a thin-walled atrial appendage, fatty infiltration of the myocardium due to myotonic dystrophy, ischemic and dilated cardiomyopathy, are variables that increase the likelihood of atrial perforation (10, 11, 13, 15, 20, 23–29).

### The mechanism and risk factors in our case

In our case, there were no apparent problems throughout the procedure. Over-screwing of the atrial lead seems unlikely because the requisite number of clockwise turns were performed under fluoroscopic guidance. Importantly, our patient had several risk factors including a history of longstanding smoking with bilateral emphysema. These risk factors increased the risk of pneumothorax. However, non-contrast chest CT raised suspicion of atrial lead perforation. Thus, we hypothesized that the atrial lead protruded through the pericardium directly into the pleural cavity, causing contralateral pneumothorax with concurrent pneumomediastinum and pneumopericardium.

### Diagnosis of lead perforation and CIED-associated pneumothorax

A patient can manifest signs and symptoms during the procedure or up to 72 hours after implantation (20). Every patient should receive a chest x-ray within 4 h post-procedure (30). Patients discharged from the hospital shortly after outpatient procedures should remain in contact with the CIED center (17).

In concerning cases, fluoroscopy, chest CT with three-dimensional reconstruction, and echocardiography assist in diagnosing lead perforation (13, 31); however, they are not as sensitive for tiny perforation (15, 19, 23, 32). ECG-gated high-resolution CT (HRCT) remains the diagnostic gold standard although the perforation may be over-diagnosed (19, 25, 27). To reduce imaging distortions caused by heart motion, prospective ECG triggering and retrospective ECG gating methods are introduced (33). Prospective ECG triggering, for instance, allows the ECG signal to regulate scanning such that projection data is only collected during diastole, which is the period of the least amount of cardiac movement. Thus, HRCT maximizes spatial resolution and results in optimal delineation of the myocardium, blood, and fat interfaces (19). HRCT also aids in lead retrieval planning because it provides a reliable estimation of the orientation of important structures around the misplaced lead (23, 32).

### The management of CIED-associated pneumothorax

**Figure 3:**
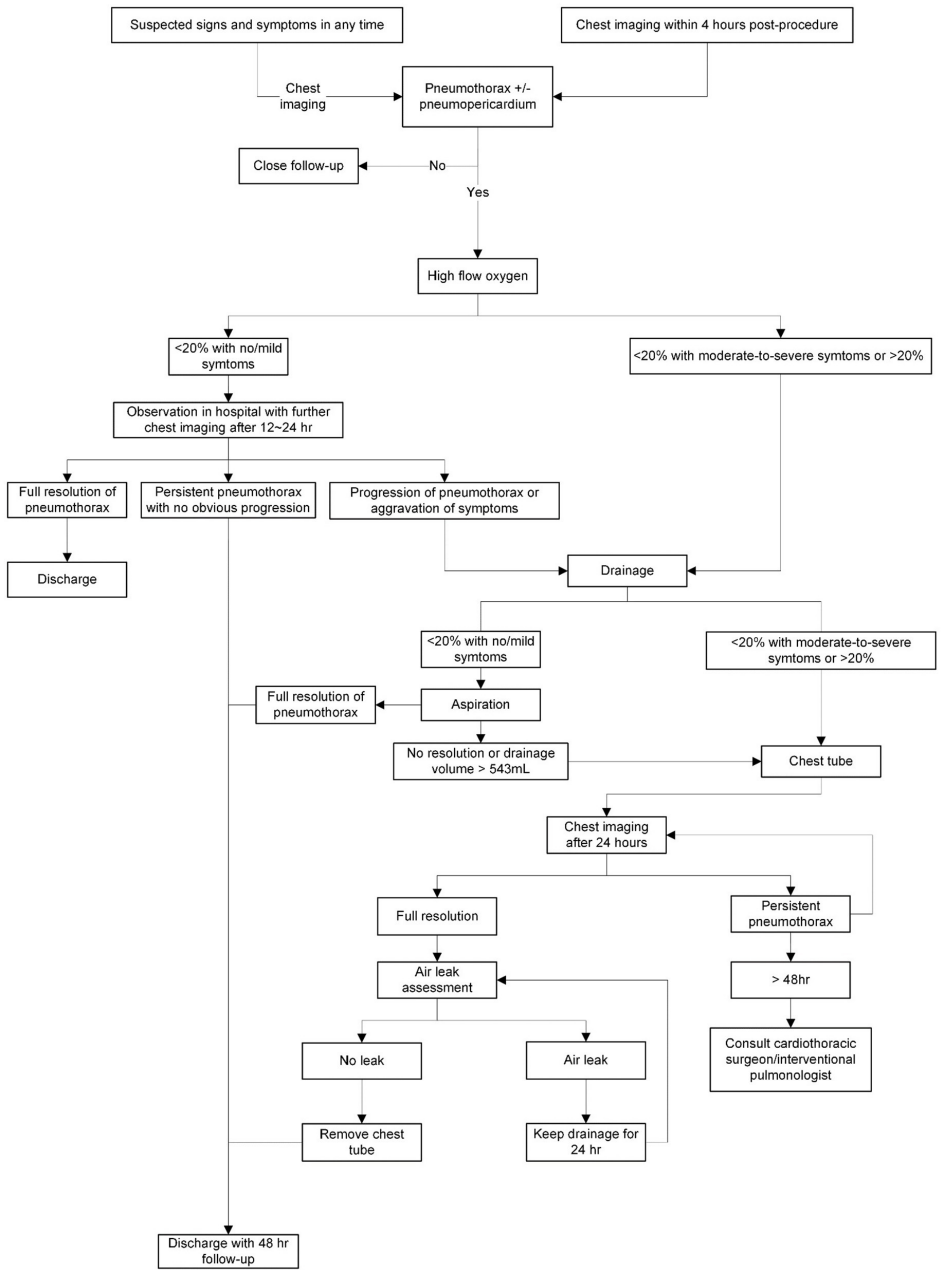
The management of cardiac implantable electronic device (CIED)-induced pneumothorax and concurrent pneumopericardium.

The management depends on the presence or absence of symptoms, the hemodynamic condition, and the extent of the lesions. Although the American College of Chest Physicians (ACCP) proposed guidelines for the management of spontaneous pneumothorax (34), which were updated by the British Thoracic Society (BTS) (35), there is no consensus on the management of iatrogenic pneumothorax, let alone CIED-induced pneumothorax. We propose a flow chart for the management of CIED-associated pneumothorax (Figure 3), which was adapted from the recommended treatment for iatrogenic pneumothorax (36).

High flow (10 L) 100% nasal oxygen, which theoretically accelerates air absorption, is generally recommended as the first step despite conflicting evidence suggesting that it has probably no effect on large pneumothoraces (13, 37, 38). A clinically stable patient with a small pneumothorax (<20%) can be observed since it may resolve on its own (12, 36). After 12–24 h, further imaging should be acquired (36). If the pneumothorax is enlarging or once symptoms worsen, drainage should be considered (36).

Needle or cannula aspiration is advocated for patients with a small (<20%) pneumothorax, minimal symptoms, and no previous parenchymal disease (36). If the patients are asymptomatic after aspiration, and repeat imaging shows resolution of the pneumothorax or no progression, they can be discharged with a 48-h follow-up (36). However, observation alone may sufficient for small iatrogenic pneumothoraces. Of note, evacuated volumes >543 mL indicate the need for further intervention with a chest tube (39).

Patients with a large (>20%) pneumothorax or those presenting moderate-to-severe symptoms should be treated with a chest tube (12–16 French) for at least 24 h (11–13, 36). If the pneumothorax improves, the absence of an air leak should be confirmed before the chest tube is removed (36). Even though a pneumothorax appears to be resolved on imaging, there may still be an air leak, which is masked by an equilibrium between the air evacuation and the air flowing into the lung through the site of puncture (36). The removal of the chest tube under this condition may lead to the reoccurrence of the pneumothorax (36).

In the majority of pneumothoraces, air leakage will stop <48 h after the placement of a chest tube (40, 41). For patients with a persistent gas leak for more than 48 h, consulting a cardiothoracic surgeon or an interventional pulmonologist is recommended (42).

### Consideration of lead extraction and repositioning

**Figure 4:**
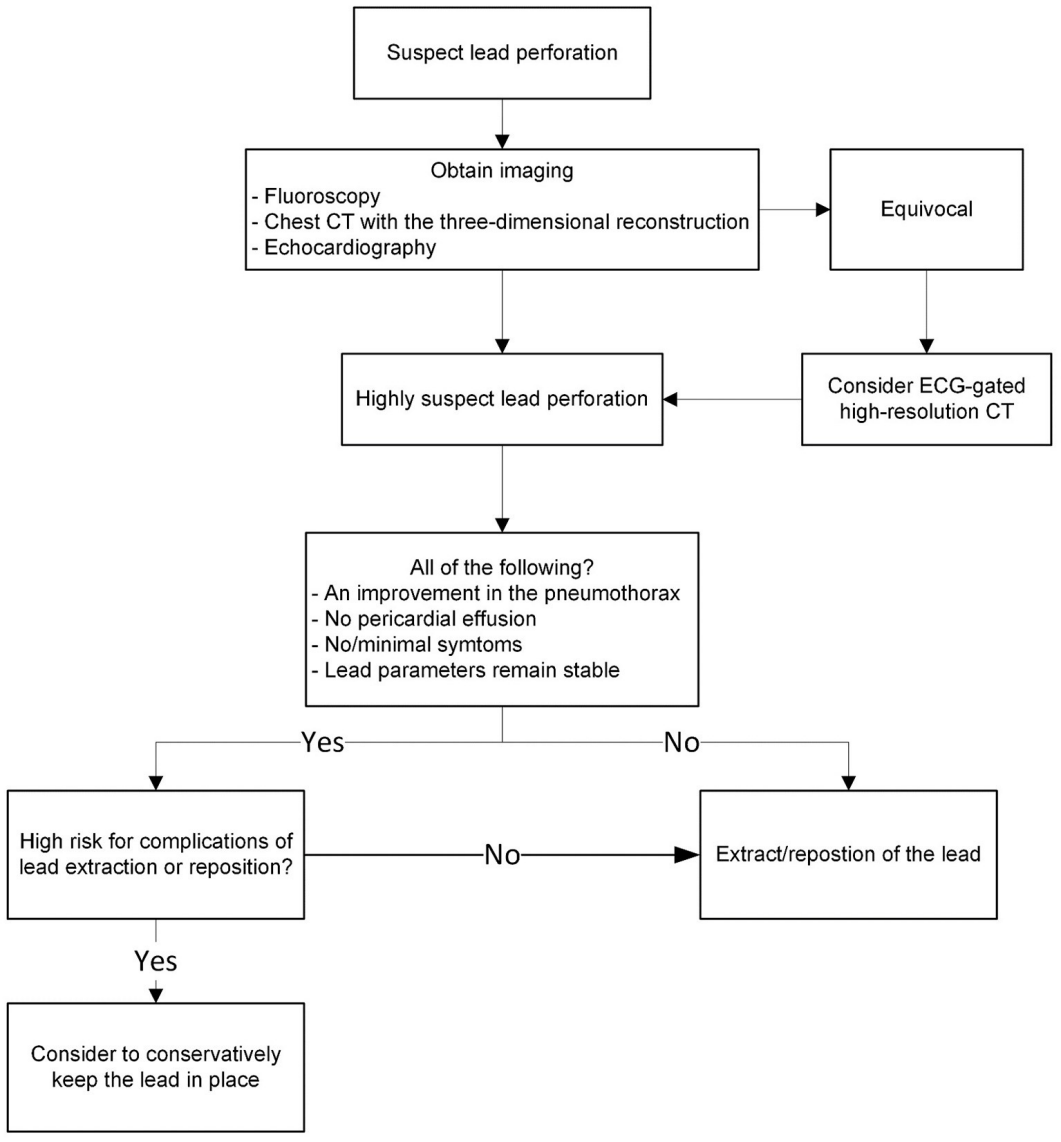
The evaluation of the need for lead extraction or repositioning.

The symptoms, imaging findings, and lead parameters are used to determine if lead extraction or repositioning is necessary (Figure 4). The lead parameters usually alter following a lead perforation (43); however, they may remain unchanged, and the patient may be asymptomatic in certain circumstances (8, 11, 13, 20, 31). The proper management of asymptomatic lead perforation is still up for debate. Despite the uncertainty, it is generally suggested the lead be extracted or repositioned because there is a chance that it will perforate the surrounding structures over time, causing catastrophic harm (27, 28, 31, 44).

Transvenous lead extraction is effective and safe management in the majority of cases. It is conducted under fluoroscopic guidance with echocardiographic and hemodynamic monitoring. Additional precautions such as placement of a pericardial drain for emergent pericardiocentesis and having cardiac surgeons on standby help assure procedural safety (15, 23, 25, 27, 28, 31, 45). Postprocedural follow-up is recommended due to the risks of constrictive pericarditis and infections (31).

Atrial lead extraction is not always required. When there is an improvement in the pneumothorax, no pericardial effusion, minimal symptoms, and satisfactory lead parameters, it is preferable to keep the electrode in place until the fibrous tissue thickens and/or wraps around the helix, especially in the elderly and weak patients who are at higher risk for complications associated with lead extraction or repositioning. It should be noted that this strategy's long-term effectiveness is unverified (11, 46).

### Prevention during venipuncture

**Figure 5:**
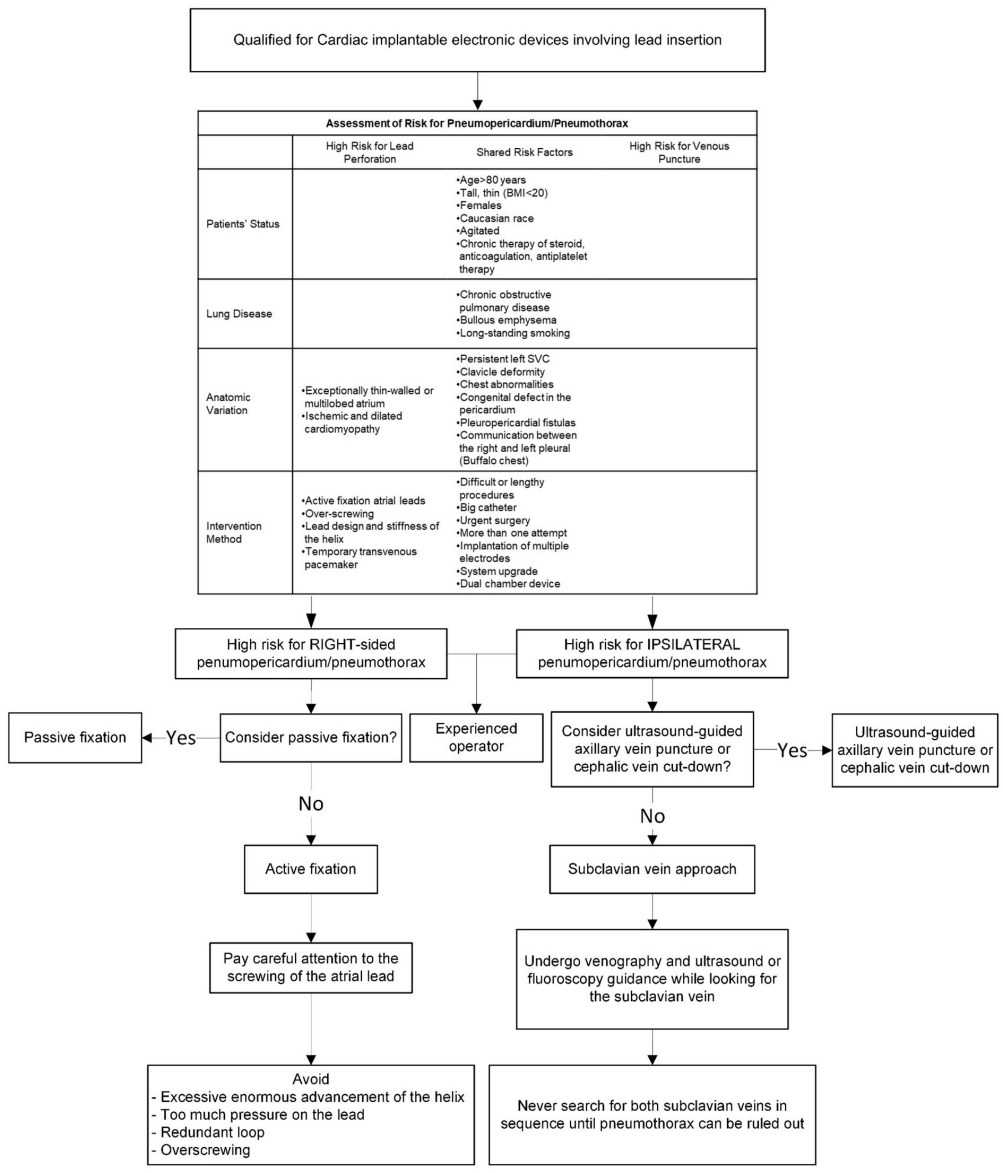
A clinical algorithm for early identification of patients at high risk of developing pneumothorax or pneumopericardium and corresponding preventive measures.

Figure 5 provides a clinical algorithm for early identification of individuals at high risk and appropriate preventative measures.

The subclavian vein has served as the most widely used venous access for CIEDs (47). In a patient with risk factors venography is recommended prior to subclavian vein puncture. Ultrasound guidance and/or fluoroscopic guidance may also be useful (48, 49). In a patient with unilateral pulmonary lesions, it's preferred to use the subclavian approach on the ipsilateral side of the diseased lung since there may be less severe complications (13). When venous access is not possible to establish, particularly after exhaustively looking for the subclavian vein on a specific side, the pacemaker-implanting physicians should rule out pneumothorax before shifting to the opposite side (13).

In terms of preventing pneumothorax, axillary venous access or cephalic vein cut-down is better than the subclavian vein approach (18, 47). The cephalic vein cut-down technique has been well-recognized for fewer occurrences of pneumothorax (50); however, the axillary approach is still not widely used due to inadequate training and a lack of familiarity (51).

Axillary vein access decreases the risk of complications due to its extra-thoracic anatomic location (52). Although “blind” puncture using anatomical landmarks is common, it is constrained by the variable relationship between the first rib and the axillary vein. About 5% of patients need a contrast-guided technique due to anatomical variations (52–54). As a result of inexperience, failed attempts, probable complications, and radiation exposure for both patients and physicians unavoidably increase (55).

Ultrasound guidance provides a direct view of the vessel, allowing the operator to monitor the needle's passage onto the subcutaneous tissue, assessing the depth of the vein, and preventing accidental arterial puncture, thus minimizing the probability of complications (52). A randomized clinical trial demonstrated that even when carried out by inexperienced operators, the ultrasound-guided axillary vein technique was much superior to cephalic vein cut-down (47). Therefore, we suggest ultrasound-guided axillary venous access as the preferred choice for CIED implantation.

## Conclusion

This case highlights the risks of pneumothorax and pneumopericardium associated with CIEDs, as well as the recommended treatment. Operators should be aware of these potential, unusual complications and precautions that can be taken to avoid them. However, there is currently no definitive guidance on the management of CIED-associated pneumothorax or pneumopericardium. We recommend more multicenter randomized, trials to compare conservative vs. invasive therapy for CIED-associated pneumothorax of varying severity and to compare the long-term outcome between conservative management and lead extraction in patients with asymptomatic lead perforation.

## Data availability statement

The raw data supporting the conclusions of this article will be made available by the authors, without undue reservation.

## Ethics statement

The study involving human participants has been reviewed and approved by National Cheng Kung University Hospital Institutional Review Board (NCKUH IRB) on Jun 28th, 2022. IRB No: A-EC-111-023. Written informed consent for participation was not required for this study in accordance with the national legislation and the institutional requirements.

## Author contributions

Conception and design, data acquisition, and critical revision of the article for important intellectual content: S-WL and J-YC. Literature review and drafting and finalizing the article: S-WL. Supervised the literature search and revision and approved the final version: J-YC. All authors contributed to the article and approved the submitted version.

## Funding

This study was supported by the Ministry of Science and Technology of Taiwan, China (MOST 110-2218-E-006-017 and MOST 110-2218-E-006-015) and Higher Education Sprout Project, Ministry of Education to the Headquarters of University Advancement at National Cheng Kung University (NCKU).

## Conflict of interest

The authors declare that the research was conducted in the absence of any commercial or financial relationships that could be construed as a potential conflict of interest.

## Publisher's note

All claims expressed in this article are solely those of the authors and do not necessarily represent those of their affiliated organizations, or those of the publisher, the editors and the reviewers. Any product that may be evaluated in this article, or claim that may be made by its manufacturer, is not guaranteed or endorsed by the publisher.
